# The analysis of different types of anorectal abscesses was conducted using the MRI 3D reconstruction technique

**DOI:** 10.1038/s41598-024-66892-3

**Published:** 2024-08-09

**Authors:** Xutao Ma, Yikun Li, Siming Xu, Bo Wang, Chen Wang

**Affiliations:** 1 Proctology Department, Longhua Hospital Shanghai University of Traditional Chinese Medicine, No.725 South Wanping Road, Shanghai, 200032 China; 2 Shanghai Shumiao Health Cloud Co. Ltd, Shanghai, China

**Keywords:** Magnetic resonance imaging, Anorectal abscess, Three-dimensional reconstruction, Sphincter impairment, Anal diseases, Anus

## Abstract

It has not yet been proven whether sepsis affects the tissue around the anal canal. To address this issue, we established three-dimensional models for various types of anorectal abscesses and utilize 3D reconstruction of Magnetic Resonance Imaging scans to assess the extent of muscle damage caused by anorectal abscesses. Patients diagnosed with anorectal abscess, selected from January 2019 to January 2022 underwent pre- and post-operative scanning of pelvic floor and perianal tissues. The aforementioned structures were segmented for the reconstruction of a three-dimensional visual model and measurement of volumes for the abscess as well as the internal and external sphincters and levator ani muscle. The study included a total of 42 patients. Three-dimensional visualization models were created for different types of anorectal abscesses, including perianal, intersphincteric, ischiorectal, and supralevator abscesses. No statistically significant differences were observed in the volume of the internal sphincter, external sphincter, and levator ani muscle between pre- and post-operative patients. The 3D model of anorectal abscess, reconstructed from MRI data, offers a precise and direct visualization of the anatomical structures associated with various types of anorectal abscesses. The infection did not result in any damage to the internal and external anal sphincter and levator ani muscle.

## Introduction

The anorectal abscess is a purulent condition that occurs in the soft tissue surrounding the rectum, and it manifests as a common acute infectious disease in humans. Around 90% of idiopathic perianal abscesses are caused by infection of the cryptoglandular glands^1^. The infection can rupture through the external sphincter, extending beyond the puborectalis or levators and spreading laterally on both sides, resulting in various types of anorectal abscesses such as perianal abscess, intersphincteric abscess, ischiorectal abscess, supralevator abscess, and even horseshoe-shaped ones^1,2^.

Typically, abscesses are categorized as either superficial or deep in relation to the anal sphincter. In case of deep abscess, imaging may be necessary for definitive diagnosis confirmation. Magnetic resonance imaging (MRI) is increasingly employed for assessing deep anorectal abscesses due to its advantages of providing a clear visualization of the abscess, high resolution of soft tissues, and absence of ionizing radiation^3^. Consequently, MRI has emerged as a crucial method for accurately determining the location of abscesses in relation to structures such as internal and external anal sphincter and levator ani muscle.

The abscesses exhibit high-intensity signal shadows in both proton density-weighted images (PDWI) and T2-weighted images (T2WI), with clear contrast between the high-signal pus of the abscess and the low-signal fibrous tissues in T2WI, enabling differentiation from normal tissues^4^. Recent studies have confirmed the utility of 3D models derived from MRI-based reconstruction in comprehending the intricate anatomical relationship between fistula tracks and normal tissues in complex anal fistulas Crohn’s fistulas^5–7^. However, it has not yet been proven whether sepsis affects the tissue around the anal canal. Despite existing research, there remains a lack of studies focusing on MRI-based three-dimensional (3D) reconstruction of anorectal abscesses and peripheral sphincteric tissue. Therefore, this study aims to demonstrate different types of anorectal abscesses and evaluate changes in volume of adjacent sphincteric tissues before and after drainage surgery using MRI-reconstructed 3D models.

## Methods

### Patients and study design

The study design involved a retrospective analysis conducted at a single center. A total of 42 patients with anorectal abscess, who were admitted to the Department of Proctology at Longhua Hospital Shanghai University of Traditional Chinese Medicine for surgical drainage treatment between January 2019 and January 2022, underwent pre-and post-operative perianal MRI scans. Patients with chronic inflammatory bowel disease, dermatitis, diabetes, tumors and pregnant or lactating women were excluded. The general exclusion criteria to MR imaging (eg, claustrophobia, pregnancy) were applicable. No other explicit inclusion or exclusion criteria were used. The study was conducted in accordance with the Declaration of Helsinki. This study was approved by the Medical Ethical Committee for Clinical Research and Animal Trials of Longhua Hospital Shanghai University of Traditional Chinese Medicine, Shanghai, China (No.: 2020LCSY032), all methods were carried out in accordance with relevant guidelines and regulations and written informed consent was obtained from all participants prior to the experiments. Privacy and confidentiality of participants was maintained throughout the study.

### Segmentation

The patients underwent examination using Philips Ingenia 3.0T MRI scanners, which utilized axial, sagittal, and coronal scanning sequences with the following parameters: T2-weighted imaging (TR/TE: 4070 ms/75 ms, section thickness:3 mm); proton density-weighted imaging (TR/TE:3500 ms/55 ms, section thickness: 3 mm). Subsequently, approximately 120 DICOM-formatted images were acquired and stored after the scanning process.

The DICOM format images acquired from the axial, sagittal, and coronal scans were imported into the open-source 3D reconstruction software ITK-SNAP 3.8. Following automatic positioning by the software, the axial position was utilized as the primary processing window for precise identification of the starting and ending positions of structures. The sagittal and coronal windows aided in localizing tissue structures that posed challenges in distinction. Manual segmentation of abscesses was performed on T2WI sequences combined with PDWI sequences using a paintbrush tool, layer by layer until their disappearance. The internal and external anal sphincter, and levator ani muscle were manually segmented on the T2WI sequence until they disappeared; different colors were assigned to represent distinct structures (Table 1). Upon completion of segmentation, basic 3D reconstruction models were obtained in the reconstruction window followed by smoothing to enhance their three-dimensional appearance.

**Table 1:** Demographic characteristics of the patients.

Structure	Color
Abscess	
Internal anal sphincter	
External anal sphincter	
Levator ani muscle	
Obturator internus	
Skin	
Femur	

### Statistical analysis

The statistical analysis was conducted using SPSS software version 25.0 (SPSS Inc., Chicago, IL, United States). First of all, the normality of the measurement data is analyzed, and the data in accordance with the normal distribution are tested by paired sample t-test. Measurement data were presented as mean ± s.d. The alpha level for all tests was set at 0.05. The tests were two-tailed. Statistical significance was considered at *p* < 0.05.

## Results

According to the different types of anorectal abscesses observed in the 42 cases, they were categorized into four groups: perianal abscess group, intersphincteric abscess group, ischiorectal abscess group, and supralevator abscess group. The demographic data can be seen in Table 2. The characteristics of abscesses and the types of surgical drainage are presented in Table 3.

**Table 2:** Patient characteristics.

Total n = 42	
Age (yr)	42.24 ± 15.13 [15–73]
Gender (male: female)	35:7
Duration (day)	10[3–7200]
Abscess type	
Perianal abscesses n (%)	4(9.52%)
The measurement of perianal abscesses (mm^3^)	7960.33 ± 6934.39
Pre- and post-operative intervals (day, median)	10.5
Intersphincteric abscesses n (%)	17(40.48%)
The measurement of intersphincteric abscesses (mm^3^)	6337.59 ± 9345.56
Pre- and post-operative intervals (day, median)	7
Ischiorectal abscesses n (%)	13 (30.95%)
The measurement of ischiorectal abscesses (mm^3^)	36,271.25 ± 31,319.37
Pre- and post-operative intervals (day, median)	15
Supralevator abscesses n (%)	8(19.05%)
The measurement of supralevator abscesses (mm^3^)	46,924.40 ± 53,277.44
Pre- and post-operative intervals (day, median)	14

**Table 3:** Comparison of pre-and post-operative volumes of perianal sphincters.

Type of surgery	Volumes (mm^3^)	Preoperative	Postoperative	*P*
Incision and drainage n = 22	Internal sphincter	4613.12 ± 1643.97	4521.38 ± 1285.13	0.620
	External sphincter	14,415.90 ± 4319.40	14,256.87 ± 4283.78	0.833
	Levator ani muscle	13,113.56 ± 3179.70	13,613.95 ± 3219.37	0.428
Catheter drainage n = 20	Internal sphincter	3884.50 ± 1157.47	3922.36 ± 1195.00	0.841
	External sphincter	12,406.29 ± 4364.88	13,049.62 ± 3044.17	0.299
	Levator ani muscle	12,294.47 ± 2881.63	13,110.16 ± 2580.42	0.192

**Figure 1:**
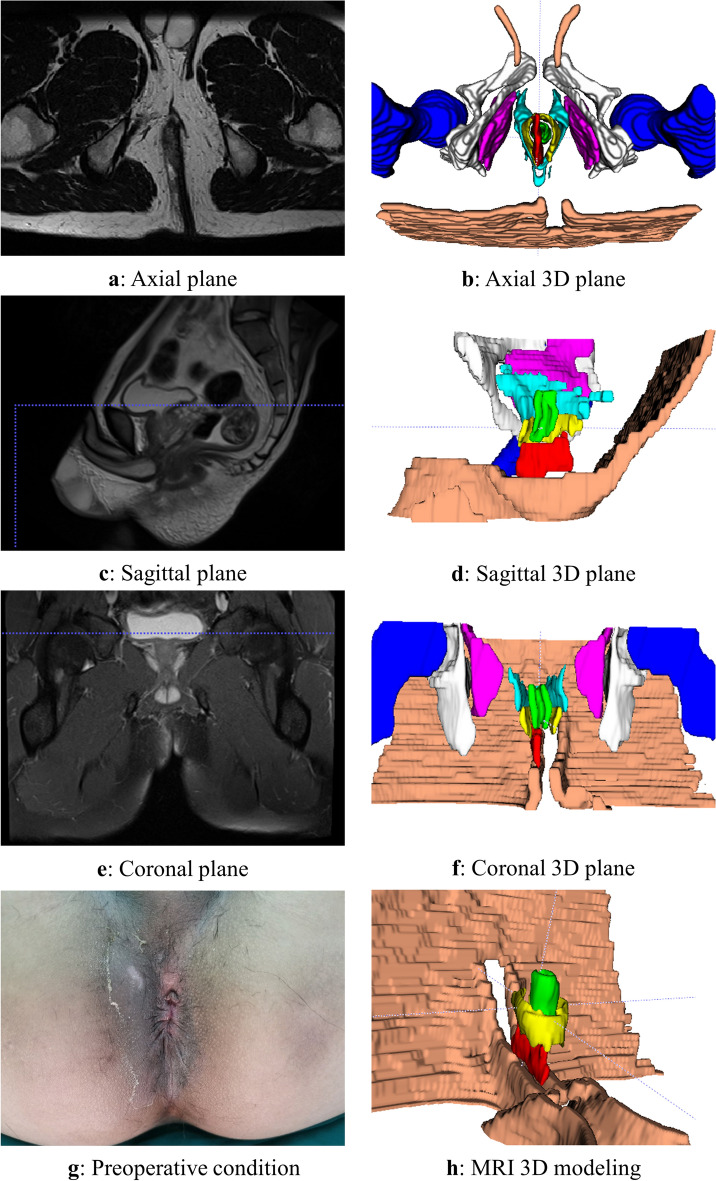
Examples show the 3D model drawing of a perianal abscess.

**Figure 2:**
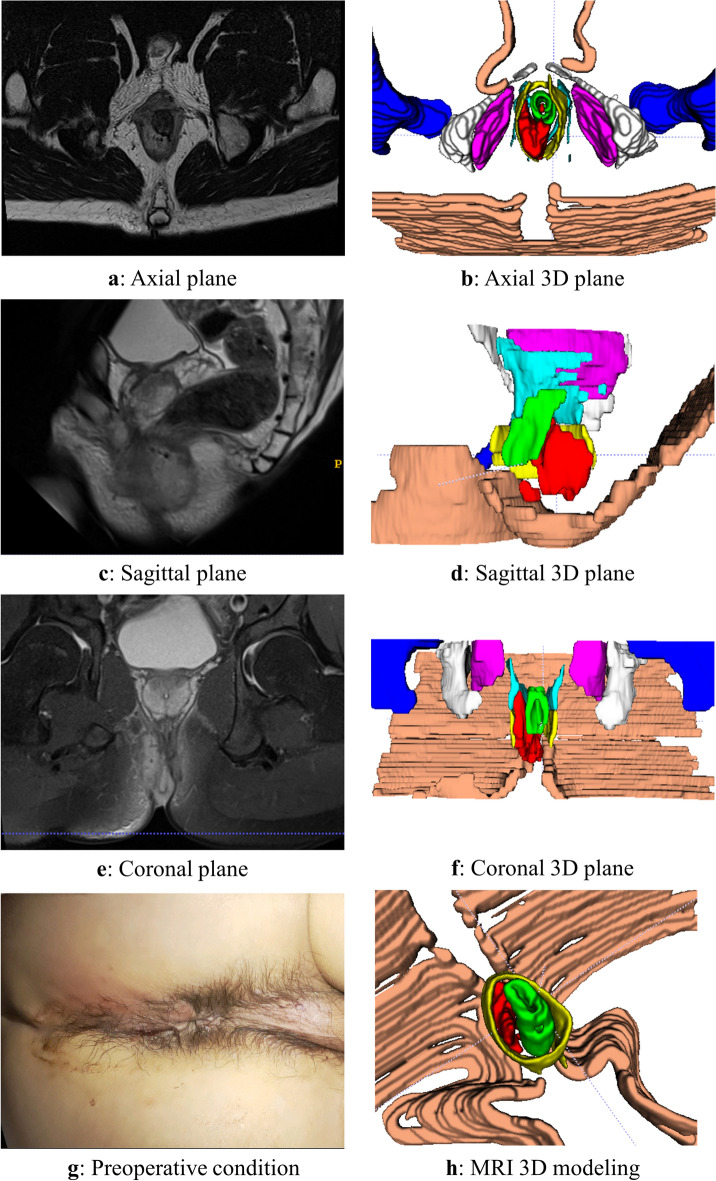
Examples show the 3D model drawing of an intersphincteric abscess.

**Figure 3:**
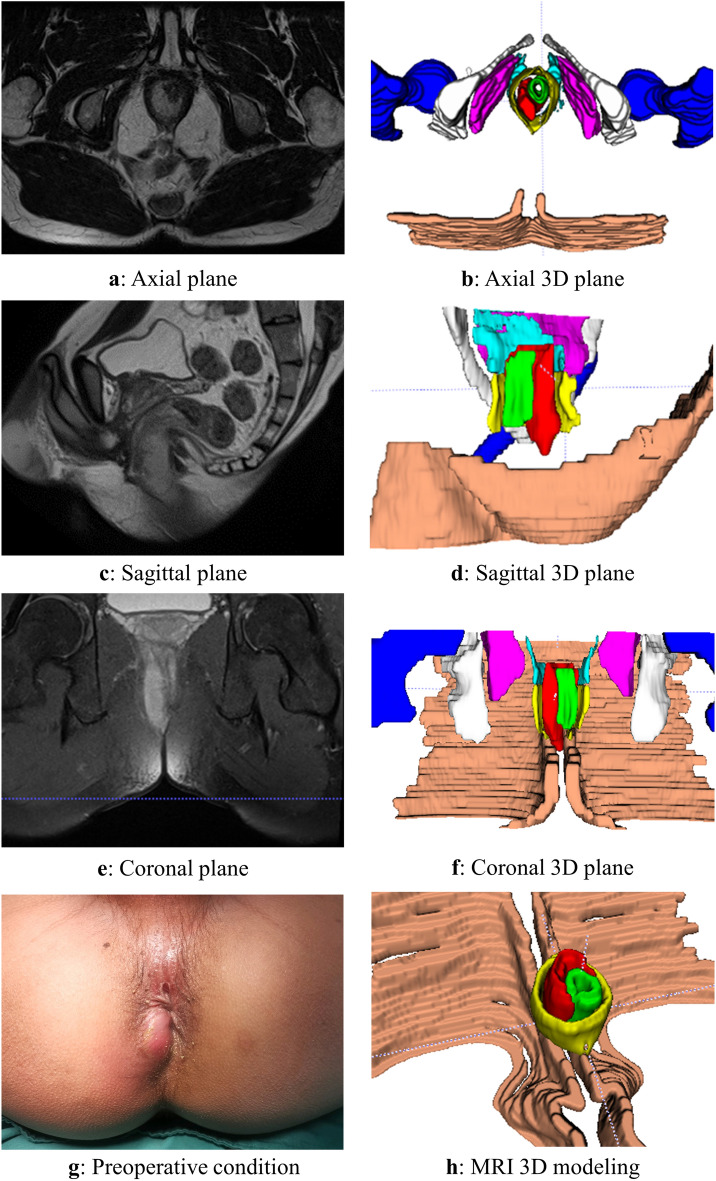
Examples show the 3D model drawing of a horseshoe intersphincteric abscess.

**Figure 4:**
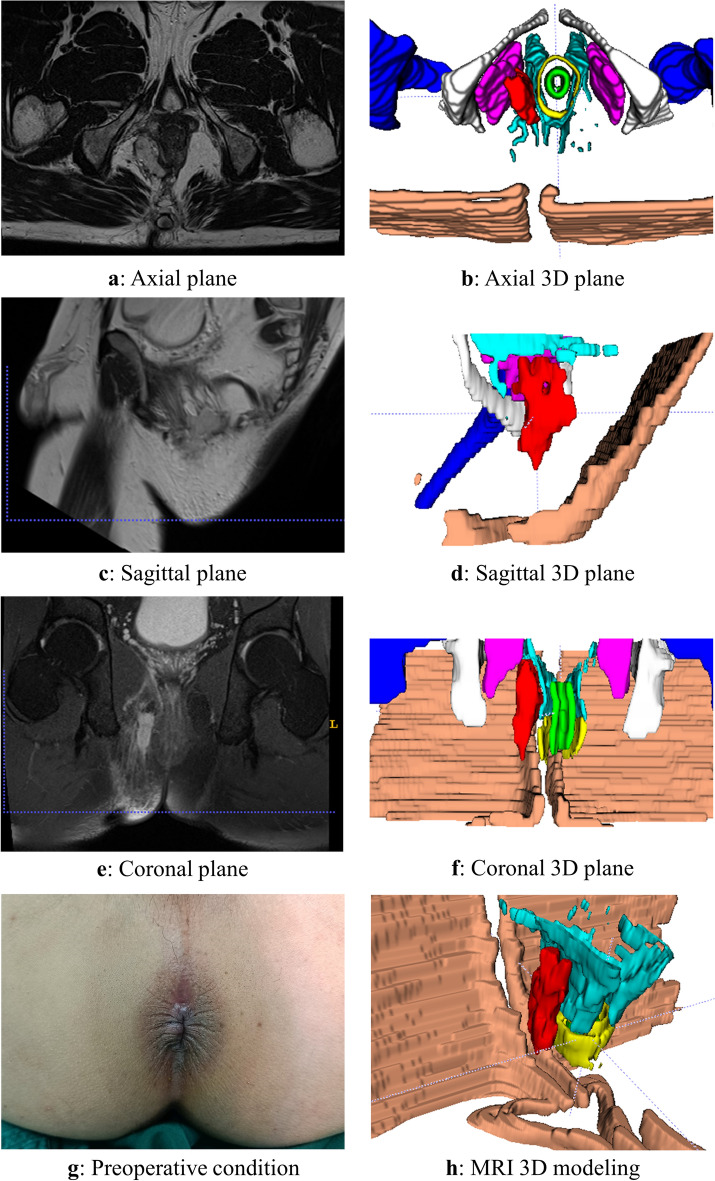
Examples show the 3D model drawing of an ischiorectal abscess.

**Figure 5:**
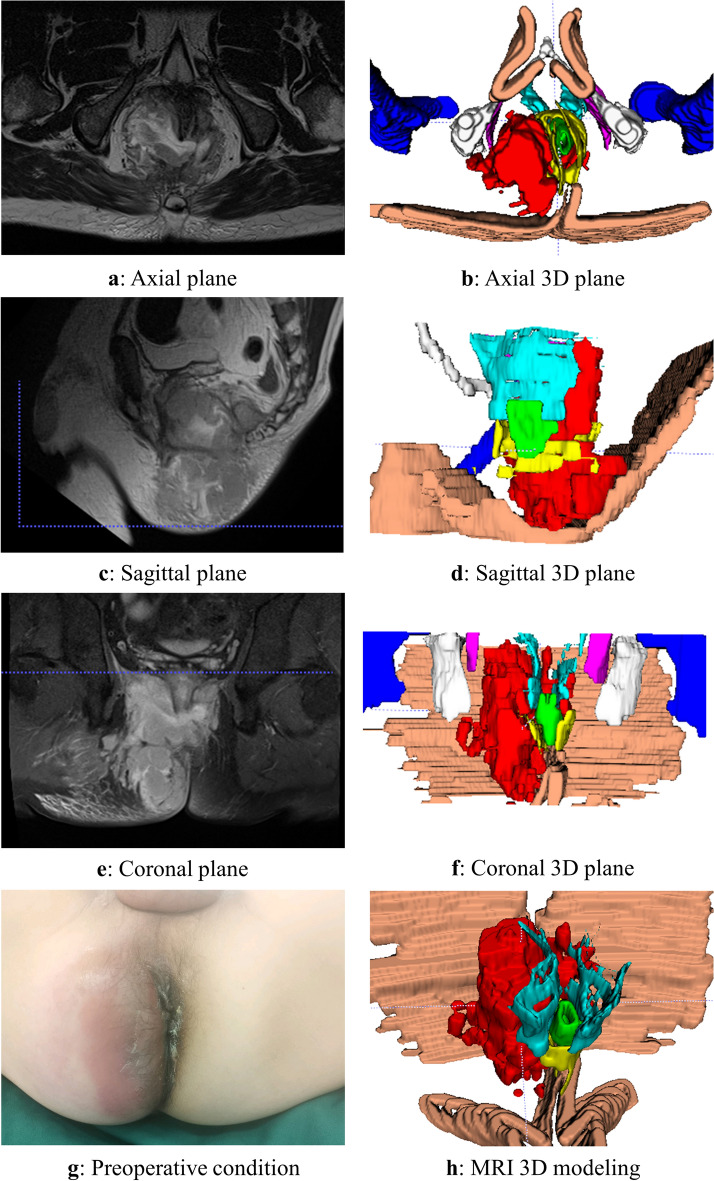
Examples show the 3D model drawing of a horseshoe ischiorectal abscess.

**Figure 6:**
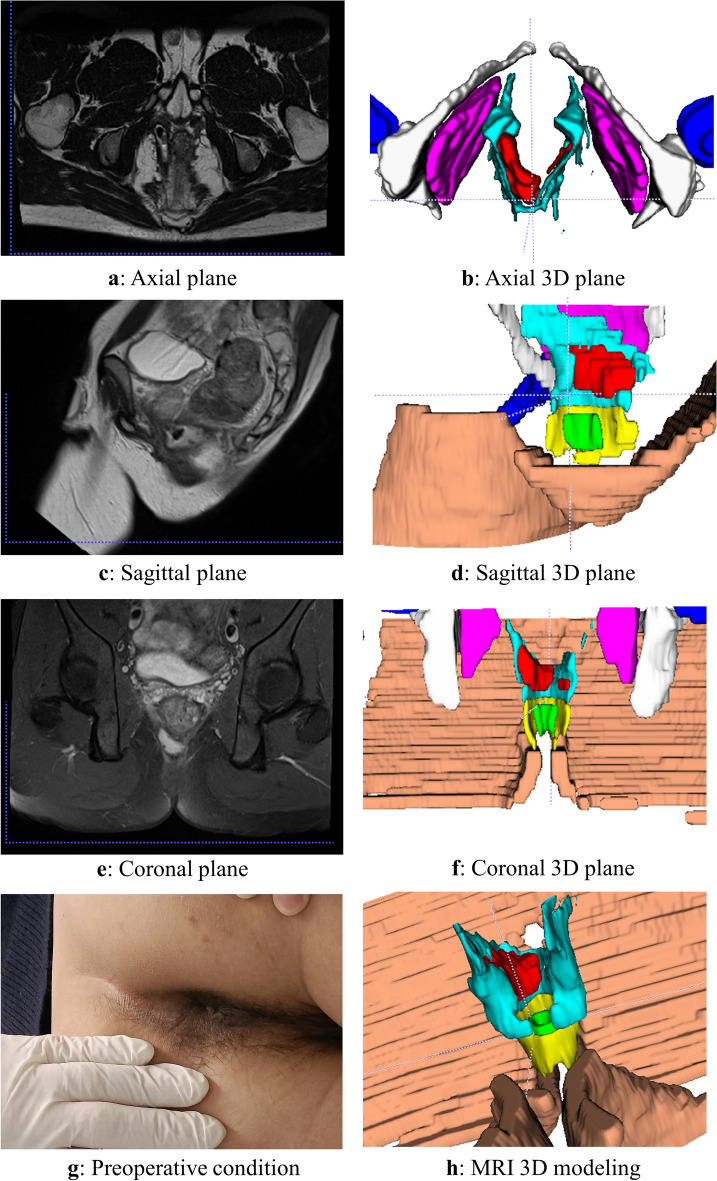
Examples show the 3D model drawing of a supralevator abscess.

The 3D reconstruction was conducted according to the aforementioned methodology, and a representative case of a typical anorectal abscess was selected for demonstration (Figs. 1, 2, 3, 4, 5, 6). The model effectively presents MRI image information simultaneously in axial, sagittal, and coronal orientations, providing functionalities such as multi-dimensional rotation, local area zooming, and selective structure hiding. These features facilitate the observation of the spatial relationship between the abscess and surrounding structures by utilizing distinct colors to represent different anatomical components. Furthermore, based on this established 3D model, data extraction from the model was performed for subsequent statistical analysis.

Based on the 3D reconstructive models, accurate measurements were conducted to determine the volumes of the internal sphincter, external sphincter, and levator ani muscle before and after the drainage operation. No significant differences were observed in the preoperative and postoperative volumes of these muscles among all patients (*p* > 0.05) (Table 4).

**Table 4:** Volume of anal sphincters at preoperative and postoperative condition.

Total n = 42	Preoperative	Postoperative	*P* value
Internal sphincter (mm^3^)	4266.16 ± 1463.14	4236.13 ± 1264.66	0.817
External sphincter (mm^3^)	13,458.94 ± 4406.48	13,681.99 ± 3750.47	0.646
Levator ani muscle (mm^3^)	12,723.52 ± 3032.83	13,374.05 ± 2908.45	0.137

The pre- and post-operative volumes of perianal sphincters were compared for different types of anorectal abscesses, and the results are presented in Table 5.

**Table 5:** Comparison of pre-and post-operative volumes of perianal sphincters.

	Volumes (mm^3^)	Preoperative	Postoperative	*P*
Perianal abscess n = 4	Internal sphincter	3393.35 ± 1111.17	3603.23 ± 1498.28	0.399
	External sphincter	10,654.36 ± 3863.64	10,841.24 ± 3944.73	0.562
	Levator ani muscle	11,074.38 ± 2849.39	13,297.95 ± 2357.60	0.093
Intersphincteric abscess n = 17	Internal sphincter	4107.00 ± 958.27	4357.79 ± 1224.26	0.233
	External sphincter	13,614.83 ± 4951.48	14,830.37 ± 5155.61	0.190
	Levator ani muscle	12,804.22 ± 3006.58	13,242.60 ± 3478.77	0.541
Ischiorectal abscess n = 13	Internal sphincter	3974.56 ± 955.49	3746.81 ± 655.78	0.420
	External sphincter	11,442.14 ± 3273.37	11,961.32 ± 2253.44	0.636
	Levator ani muscle	12,561.37 ± 4244.59	12,824.02 ± 2876.58	0.862
Supralevator abscess n = 8	Internal sphincter	3974.56 ± 955.49	3746.81 ± 655.78	0.420
	External sphincter	11,442.14 ± 3273.37	11,961.32 ± 2253.44	0.636
	Levator ani muscle	12,561.37 ± 4244.59	12,824.02 ± 2876.58	0.862

## Discussion

An anorectal abscess is a localized collection of infected fluid around the anal canal. The American Society of Colon and Rectal Surgeons clinical practice guidelines for the management of anorectal abscess suggested that patients with acute anorectal abscess should be treated promptly with incision and drainage^8^. During the surgery, we made the incision large enough to provide adequate drainage while taking care not to injure the anal sphincter complex and surgical drains (eg, T-tube, Mushroom) were placed into the abscess cavity to provide adequate drainage in case with horseshoe shape or deep abscess cavity. Typically, pelvic muscle measurements are based on imaging figures judged by doctors for grading^9–12^. This study utilizes 3D reconstruction models to quantitatively measure the volume of abscess cavities and surrounding muscular tissues. The obtained volume from muscular measurements can also aid in assessing whether sepsis infection has destroyed perianal sphincteric tissue.

The MRI images revealed that the majority of anorectal abscesses disseminated from lesions located between the sphincters, following the path of loose connective tissues within the interspaces. This dissemination process involved liquefaction of connective tissues and subcutaneous fat, resulting in localized areas exhibiting high signal intensity and distinct contrast compared to surrounding tissues.

Interestingly, in the case of extensive abscess lesions such as horseshoe-shaped abscesses (as shown in Fig. 5), MRI images revealed involvement of the sphincteric muscle, resulting in poorly displayed images. However, 3D reconstruction models demonstrated the abscess and surrounding tissue more clearly. The findings of this study indicate that there was no reduction in the volume of either the sphincter or levator muscle around the anorectal abscess. Regarding intersphincteric abscesses, infection between the internal and external sphincters caused liquefaction of tissue on MRI images. Some external sphincters appeared thickened and swollen to varying degrees, showing a higher signal on T2WI sequences that was poorly demarcated from the abscess. It has been suggested that infection at post anal canal space may spread to the muscle to form an abscess, providing a reference for intersphincteric abscess formation; however, there is no unanimous agreement on this mechanism and further confirmation is needed through subsequent studies^13^.

After utilizing 3D reconstruction software to refine and repair the research object, a meticulously crafted anorectal abscess model with distinct anatomical structure is obtained in this study, allowing for convenient selection and rotation. However, the current reliance of 3D reconstruction software on imaging data such as MRI or CT necessitates manual or semi-automatic methods to identify and delineate structures. The clarity of the MRI image and the researcher’s discernment significantly influence the consistency and precision of the 3D reconstruction.

However, it is important to note that this retrospective study included all patients who had undergone the MRI examination. In comparison to endoanal MRI or endorectal ultrasound, employing a phased array coil for MRI offers a non-invasive approach that can effectively alleviate patient discomfort.

There are certain limitations associated with this study. Firstly, it should be noted that this is an exploratory study focused on the 3D reconstruction technique of various anorectal abscesses. Therefore, we have only included a sample size of 42 cases to establish correlations. In future studies, we plan to expand our sample size for further verification. Secondly, the intervals between pre-and post-operative perianal MRI examinations were varied among patients, possibly impacting the accuracy of abscess volumes as well as those of internal and external anal sphincter and levator ani muscle. Finally, it is insufficient to solely rely on the volume of muscle reconstructed from MRI data to prove comprehensive results. Although the MRI reconstruction showed no damage to the internal and external anal sphincter and levator ani muscle in this study, future research will incorporate Wexner anal function score and anorectal manometry to evaluate sphincter damage.

## Conclusions

The present study demonstrates 3D models of perianal abscess, intersphincteric abscess, ischiorectal abscess, and supralevator abscess based on MRI reconstruction. Moreover, our findings indicate that the infection did not result in any damage to the internal and external anal sphincter and levator ani muscle. Additional investigations with larger sample sizes, more precise modeling techniques, and a standardized research plan are imperative to validate our results.

### Supplementary Information

Supplementary Information.

## Data Availability

All data generated and analyzed during this study are available from the corresponding author.

## References

[1] Eisenhammer, S. The internal anal sphincter and the anorectal abscess. *Surg. Gynecol. Obstet.* **103**, 501–506 (1956). 13360660

[2] Whiteford, M. H. Perianal abscess/fistula disease. *Clin. Colon. Rectal. Surg.* **20**, 102–109 (2007). 20011384 10.1055/s-2007-977488 PMC2780182

[3] Maruyama, R. *et al.* Usefulness of magnetic resonance imaging for diagnosing deep anorectal abscesses. *Dis. Colon. Rectum.* **43**, S2–S5 (2000). 11052470 10.1007/BF02237218

[4] Fukuzako, S. *et al.* Perirectal abscess with dysuria. *JGH Open.* **4**, 548–549 (2020). 32514469 10.1002/jgh3.12307 PMC7273732

[5] Lam, D., Yong, E., D’Souza, B. & Woods, R. Three-dimensional modeling for crohn’s fistula-in-ano: A novel interact approach. *Dis. Colon. Rectum.* **61**, 567–572 (2018). 29624551 10.1097/DCR.0000000000001084

[6] Sahnan, K. *et al.* Improving the understanding of perianal. crohn fistula through 3D modeling. *Ann. Surg.* **267**, e105–e107 (2018). 29232211 10.1097/SLA.0000000000002629

[7] Day, N. J., Earnshaw, D., Salazar-Ferrer, P. & Walsh, C. J. Preoperative mapping of fistula-in-ano: a new three-dimensional MRI-based modelling technique. *Colorectal. Dis.* **15**, e699–e701 (2013). 24119050 10.1111/codi.12438

[8] Wolfgang, B. G. *et al.* The American Society of Colon and Rectal Surgeons Clinical Practice Guidelines for the Management of Anorectal Abscess, Fistula-in-Ano, and Rectovaginal Fistula. *Dis. Colon Rectum.* **65**, 964–985 (2022). 35732009 10.1097/DCR.0000000000002473

[9] Zbar, A. P. *et al.* Use of vector volume manometry and endoanal magnetic resonance imaging in the adult female for assessment of anal sphincter dysfunction. *Dis. Colon. Rectum.* **42**, 1411–1418 (1999). 10566528 10.1007/BF02235038

[10] Lammers, K., Prokop, M., Vierhout, M. E., Kluivers, K. B. & Fütterer, J. J. A pictorial overview of pubovisceral muscle avulsions on pelvic floor magnetic resonance imaging. *Insights. Imaging.* **4**, 431–441 (2013). 23756995 10.1007/s13244-013-0261-9 PMC3731469

[11] DeLancey, J. O., Sørensen, H. C., Lewicky-Gaupp, C. & Smith, T. M. Comparison of the puborectal muscle on MRI in women with POP and levator ani defects with those with normal support and no defect. *Int. Urogynecol. J.* **23**, 73–77 (2012). 21822711 10.1007/s00192-011-1527-8 PMC3508430

[12] Zhuang, R. R. *et al.* Levator avulsion using a tomographic ultrasound and magnetic resonance-based model. *Am. J. Obstet. Gynecol.* **205**(232), e1-232.e8 (2011). 10.1016/j.ajog.2011.03.052 21620359

[13] de Miguel Criado, J. *et al.* MR imaging evaluation of perianal fistulas: Spectrum of imaging features. *Radiographics* **32**, 175–194 (2012). 22236900 10.1148/rg.321115040

