# Supplementary material for: The analysis of different types of anorectal abscesses was conducted using the MRI 3D reconstruction technique
Source: Sci Rep. 2024 Aug 9;14:18473. doi: 10.1038/s41598-024-66892-3 (PMC11316023; doi:10.1038/s41598-024-66892-3)
Supplement: Supplementary file 1 — Supplementary Information. [file 41598_2024_66892_MOESM1_ESM.pdf]

The analysis of different types of anorectal abscesses was conducted using the MRI 3D reconstruction technique

Xutao Ma, [1] Yikun Li,[1] Siming Xu,[1] Bo Wang[2] & Chen Wang[1*]

1 Proctology Department, Longhua Hospital Shanghai University of Traditional Chinese Medicine, Shanghai, China

2 Shanghai Shumiao Health Cloud Co. Ltd, Shanghai, China

Correspondence: Chen Wang, M.D., Proctology Department, LongHua Hospital Shanghai University of Traditional Chinese Medicine, No.725 South Wanping Road, Shanghai 200032, China. E-mail: wangchen_longhua@163.com

		Volume of abscess		Volume of Internal sphincter		Volume of External sphincter		Volume of Levator ani muscle	
Gender	Abscess type	Preoperative(mm^3)	Postoperative(mm^3)	Preoperative(mm^3)	Postoperative(mm^3)	Preoperative(mm^3)	Postoperative(mm^3)	Preoperative(mm^3)	Postoperative(mm^3)
1	2	18250.6	4427.22	4499.97	5541.21	14244.5	14100.9	14167.9	12131.3
1	3	12167.7	7420.84	5463	4930.64	18047.7	13253	21282.4	14602.4
1	1	1883.44	618.243	4111	3414.69	11492.1	12571.6	16158.6	17442.9
1	2	24455.9	32709.6	3880	3757.85	10124.2	13717.3	14276.8	13410.9
1	1	29633.5	1544.65	6632.22	5129.69	13704.7	16237	12137.1	12650
1	0	2934.26	1567.62	3617.58	3818.56	10640.3	10904.4	9298.52	10376.1
1	2	20106	2145.67	4039.07	5845.56	17272.9	16510.7	10942.1	10519.7
1	1	4063.55	574.219	9453.55	8016.09	20555.1	13890.3	13924.8	15842.7
1	1	4844.5	512.968	4945.95	4153.51	15226.4	12512.2	15674.3	13643.4
2	2	24253	6829.37	4706.67	4385.11	14366.9	14642.6	16503	19360.7
1	3	27340.5	1997.8	3158.21	3474.72	11524.6	8845.85	9526.31	6677.84
2	0	1974.27	445.977	2579.04	2790.71	6389.96	7011.22	9002.19	12420.4
2	2	62545.6	24105.7	3014.64	3625.23	8726.18	10808.7	15928.8	15898.2
1	1	212.461	495.742	3548.68	4310.47	14275.1	15475.2	11128.4	6974.84
1	2	19990.4	1171.41	2333.24	2455.74	4978.46	7591.18	12634.7	12847.2
1	1	2859.61	1376.21	6232	4706.68	18845.9	16570	13388.9	13231.9
2	2	12431.8	817.305	2407.88	2229.88	7411.23	9589.45	11955.2	9779
1	3	102806	1875.78	3949.14	2901.72	6212.51	9053.52	6603.62	12318.9
1	3	26613.1	99.5315	4013.78	3682.66	11203	10552.2	12424.2	11664.3
1	1	3003.16	586.932	2884.49	4691.77	10358.9	14444.4	13666.4	17035.8
2	1	3047.19	669.921	2880.67	3537.18	9501.43	15697.2	15787.2	14742.1
1	1	5160.5	1349.42	4409.06	5033.99	15874.4	15364.2	10646.9	14772.8
2	0	16822.7	5273.24	2501.67	2191.6	9840.17	9195.15	10818.3	14923.9
1	1	1843.24	570.39	5091	4209	17307	11968.6	12810.8	17096.4
2	3	6820.93	0	2742.62	3521.87	10101.9	13195.5	13640.7	14240.6
1	1	411.632	243.086	1481.15	1649.92	6159.38	7351.91	9460.89	9855.5
1	1	8572.45	468.945	4295.16	5633.08	16951	17574.9	14694.9	14770.8
1	1	3573.29	461.29	6142.51	5156	17656	14307.6	16157.3	16020.7
1	3	18819.2	602.929	3152.43	3311.32	10920.4	14074.1	13016.5	13325.7
1	2	60244.4	677.579	4604.01	4027.2	15618.5	11995.5	11316.9	12158.1
1	2	120861	8970.24	5200.5	6502.7	21542.8	28598.9	13094.1	19934.6
1	3	24985.8	5167.97	4224.36	4499.97	12680.9	14744	13288.7	13354.4
1	1	126.328	61.25	2823.25	2528.48	10284.3	11832.7	6800.67	11972.5
1	1	31450.8	3477.31	6375.06	6031.76	19424.2	15987.5	12026.6	11436
1	2	17580.7	2313.03	4125.38	4026.71	13448.7	18437.8	5191.27	8112.64
1	2	13000.3	5945.08	4659	4775.59	20773.3	16545.2	14738.3	13032.9
1	2	58166.5	2183.1	4863.64	4591.93	16669.6	16636.1	15381.4	14278.6
1	0	10110.1	2637.58	4875.12	5612.03	15747	16254.2	15178.5	15471.4
1	3	155842	1419.59	5092.9	3651.58	10846.1	11972.4	10708.5	16408
1	1	2649.05	645.04	3389.02	3577.39	16020.4	15130.7	14401.7	12177.3
1	1	4404.25	1558.04	5723.04	5099.05	20491.9	15877.1	14278.9	14106.6
1	2	19640.1	1755.2	5056.94	4886.6	11815.5	13620.5	10324.4	10690
